# An unexpected ally in heart failure treatment: Unlocking the potential of sodium‐glucose cotransporter 2 inhibitors across patient subpopulations

**DOI:** 10.1002/ehf2.15167

**Published:** 2024-11-16

**Authors:** Sander Trenson, Mateusz Sokolski

**Affiliations:** ^1^ Department of Cardiology Sint‐Jan Hospital Bruges Bruges Belgium; ^2^ Faculty of Medicine, Institute of Heart Diseases Wroclaw Medical University Wroclaw Poland; ^3^ Institute of Heart Diseases University Hospital Wroclaw Poland

Heart failure (HF), a leading cause of morbidity and mortality, globally affects millions of people. Sodium‐glucose cotransporter 2 inhibitors (SGLT2i), originally developed as antihyperglycaemic drugs, have shown broad therapeutic efficacy in managing HF across the spectrum of ejection fractions. Recent research explored evidence on efficacy and safety of SGLT2i across HF subtypes, focusing on their mechanisms, clinical benefits and implications for future HF management.

SGLT2i, including dapagliflozin and empagliflozin, work by decreasing glucose reabsorption in the proximal renal tubule, promoting glucosuria and mild osmotic diuresis. This mechanism contributes to improved glycaemic control and a lower body weight. Importantly, the beneficial effects of SGLT2i on HF are irrespective of diabetes mellitus or other co‐morbidities, with robust results on mortality, HF hospitalisations and renal function.^1^, ^2^

Real‐world nationwide data in >50 000 patients from the Swedish HF population confirmed the compelling evidence from the randomized trials that patients with HF who receive SGLT2i were at significant lower risk for all‐cause and cardiovascular mortality and HF and all‐cause readmissions. The results were consistent in the overall and diabetes cohorts. The effect size was greater than in randomized trials.^3^ Different explanations may be valid, as the event rates are often much higher in real‐world than those reported in the randomized trials, the latter including more stable HF patients with fewer or less severe co‐morbidities.

Not only is the efficacy of SGLT2i well‐established, but their use has furthermore proven to be safe. A large contemporary meta‐analysis of randomized controlled trials with over 20 000 participants could not only show reduced mortality but also show reduced severe adverse events. The effect was more prominent in patients with reduced ejection fraction and patients taking dapagliflozin. Next, renal events were reduced, and there were no discontinuations due to major hypoglycaemia, ketoacidosis or adverse limb events. However, the risk of genitourinary infections and volume depletion was higher compared with placebo, emphasizing the need for thorough patient education when initiating SGLT2i therapy.^4^

It remains an open question as to whether all SGLT2i are equally effective for the treatment of HF. Head‐to‐head comparisons of SGLT2i treatments in HF are lacking. A network meta‐analysis based on a Bayesian statistical approach revealed no significant difference among different SGLT2i treatments for the composite outcomes and individual clinical endpoints in patients with HF. By ranking probability, sotagliflozin had the lowest risk of the composite of cardiovascular death or HF hospitalization, whereas dapagliflozin had the lowest risk of all‐cause mortality and cardiovascular death, regardless of ejection fraction.^5^ Another meta‐analysis revealed dapagliflozin to have a stronger protective effect in preventing mortality without a significant difference in serious adverse events.^4^ The results should be interpreted with caution because of the heterogeneity between the included randomized clinical trials and other sources of potential biases.

Another area that requires further clarification is the precise mechanism by which these drugs exert their effects. Cardiac remodelling in HF involves structural and functional myocardial changes, typically fibrosis, endothelial dysfunction, inflammation and shifts in cellular composition and metabolics, leading to reduced cardiac function and disease progression.^6^, ^7^, ^8^ Meanwhile, the effects of SGLT2i extend beyond glycemic control and diuresis by promoting glucosuria. Mechanistic studies of cardiac remodelling demonstrated that SGLT2i intake induces regression of left ventricular mass in patients with type 2 diabetes or coronary artery disease, as shown by Verma et al. in the EMPA‐HEART CardioLink‐6 trial.^9^ Substudies of this randomized trial with empagliflozin suggest that SGLT2i mitigate modulation of inflammatory and profibrotic responses. Patients with an elevated baseline neutrophil‐to‐lymphocyte ratio and insulin‐like growth factor‐binding protein 7 levels exhibit a significantly higher left ventricular mass, but empagliflozin treatment was consistently beneficial on LV reverse remodelling irrespective of these marker levels.^10^, ^11^ Moreover, the reduction in left ventricular mass was irrespective of the duration of diabetes.^12^ These findings must be interpreted with consideration of small sample sizes and *post hoc* exploratory study designs. Nevertheless, they make us curious about the pivotal effects SGLT2i might have in cardiac remodelling.

Chronic kidney disease (CKD) is one of the most common co‐morbidities that increase morbidity and mortality in HF.^13^ A meta‐analysis by Kato et al. showed that the beneficial effect of SGLT2i is similar in HF patients with and without CKD.^14^ There are also reports on the use of SGLT2i in patients with end‐stage renal disease on haemodialysis, demonstrating that, when used together with sacubitril/valsartan, it is both effective and safe.^15^ The mechanism of action of SGLT2i through proximal renal tubules may also affect kidney health. In cases of acute decompensated HF, empagliflozin caused significant reduction in markers of tubular kidney damage within just 3 days of use. A potential mechanism of this renoprotective effect is a regional haemodynamic change of at the kidney level, leading to reduced glomerular filtration pressure without altering systemic general haemodynamic parameters, such as cardiac output and systemic vascular resistance index.^16^ However, Dai et al. demonstrated in an animal model that dapagliflozin reduced infiltration, fibrosis, and maladaptive remodelling of the right atrium and pulmonary arteries, lowering the risk of right heart dysfunction and indirectly susceptibility to atrial fibrillation.^17^ This mechanism may be significantly beneficial in patients with advanced HF who are candidates for heart transplantation, as it helps maintain low pulmonary vascular resistance, a known risk factor for right ventricular failure of the graft.^18^ This also applies to less‐studied causes of HF, such as congenital heart disease. Egorova et al. used SGLT2i in patients with congenitally corrected transposition of the great arteries and a systemic right ventricle, who could not tolerate sacubitril/valsartan. Treatment with SGLT2i resulted in less HF hospitalizations and improvements in functional and echocardiographic status.^19^

Although there is ample evidence for conventional HF medications, the role of the four pillars of HF therapy in different HF subpopulations remains to be fully elucidated. Strict inclusion criteria often exclude complex patient populations from randomized trials. For example, in patients with transthyretin amyloid cardiomyopathy, therapeutic response on angiotensin‐converting enzyme inhibitors and beta blockers seems to be different from other patients with HF.^20^ Therefore, neurohormonal blockers are currently not widely prescribed and often discontinued in transthyretin amyloid cardiomyopathy.^21^ Initiation of dapagliflozin seemed to be well tolerated in patients with tafamidis‐treated transthyretin amyloid cardiomyopathy. Observational data indicate potential improvement in biomarker levels, suggesting a role in disease stabilization.^22^ However, patient groups were small and the study was retrospective. Moreover, mouse models could not show a benefit for dapagliflozin in the progression of heart failure, cardiac inflammation and pathological changes in transthyretin amyloid cardiomyopathy.^23^ Collaborative prospective trials to expand sample sizes are highly needed.

Reports on the cost‐effectiveness of using SGLT2i across the spectrum of HF are also noteworthy. Tang et al. assessed the utility of dapagliflozin in HFpEF/HFmrEF from the perspective of the Chinese public healthcare system, demonstrating its effectiveness based on the total cost, quality‐adjusted life‐years per patient, and the incremental cost‐effectiveness ratio.^24^ Similarly, Tsutsui et al. demonstrated the cost‐effectiveness of empagliflozin in HFpEF in Japan.^25^ Extending this analysis to European countries, Kolovos et al. confirmed that empagliflozin is both clinically and cost‐effective for patients with HFpEF/HFmrEF, supporting its use across diverse healthcare environments.^26^

In summary, SGLT2i have transformed to a foundational pillar of HF treatment over the recent years, offering therapeutic benefits across the HF spectrum regardless of diabetic status. Research suggested multifactorial mechanisms of action, encompassing among others glycaemic control, increased diuresis, blood pressure reduction, renal protection and cardiac remodelling by modulation of inflammatory and profibrotic pathways. SGLT2i deservedly earned their position as a class I recommendation for HF. With ongoing studies and real‐world data supporting their broad utility and safety, clinicians and researchers will continue to optimize their use to tackle the persistent challenges in HF management.

## Conflict of interest statement

ST reports speaker fees/travel support from Boehringer Ingelheim, AstraZeneca, Bayer, NovoNordisk, Menarini and Servier. MS does not report conflicts of interest.
